# The Mediator Role of Aggression in the Relationship between Marital Stress and Depression among Patients with Coronary Artery Disease

**DOI:** 10.21315/mjms2019.26.4.11

**Published:** 2019-08-29

**Authors:** Mozhgan Saeidi, Ali Zakiei, Saeid Komasi

**Affiliations:** 1 Cardiac Rehabilitation Center, Kermanshah University of Medical Sciences, Kermanshah, Iran; 2 Sleep Disorders Research Center, Kermanshah University of Medical Sciences, Kermanshah, Iran; 3 Clinical Research Development Center, Imam Reza Hospital, Kermanshah University of Medical Sciences, Kermanshah, Iran; 4 Lifestyle Modification Research Center, Kermanshah University of Medical Sciences, Kermanshah, Iran

**Keywords:** aggression, cardiovascular diseases, depression, marital conflict, rehabilitation

## Abstract

**Background:**

Depression is one of the most important consequences of cardiovascular diseases (CVDs), and to control and treat it, it is necessary to identify its direct and indirect triggers and underlying factors. Therefore, the current study aims to evaluate and investigate the mediator role of aggression in the relationship between marital stress and depression.

**Methods:**

The sample of current cross-sectional study includes 212 patients with coronary artery disease (CAD) in Iran evaluated from Jan to Jun 2017. The required data were gathered using Beck’s Depression Inventory (BDI) questionnaire, Buss and Perry’s Aggression Questionnaire (BPAQ), and Hudson’s Marital Satisfaction Index (HMSI). The data were analysed using Pearson’s correlation coefficient and structural equation modeling (SEM) using SPSS20 and AMOS software.

**Results:**

The mean age of participants (68.4% male) was 58.5 ± 8.9. The results show that there is a significant positive relationship between all the variables (*P* < 0.05). The results of the model show that marital stress cannot directly predict depression (*P* = 0.586). However, through aggression, marital stress can significantly predict 18% of the variance of depression (*P* < 0.001).

**Conclusions:**

Not directly, but indirectly through aggression, marital stress can significantly predict increased depression among patients with CAD. The physiological and psychological pathways of the findings can be discussed.

## Introduction

Depression is considered as one of the most important consequences of cardiovascular diseases (CVDs) since it can increase the mortality rate among these patients by 20% (1–3). In order to control and treat the depression caused by a cardiovascular event, the underlying triggers and factors must be identified (1). Previous studies have highlighted the role of factors such as life events (4), socio-demographic factors and poor social support (1, 5), personality traits (4), risk factors such as smoking, history of diabetes, high cholesterol levels, and sedentary lifestyle (1, 4, 5), comorbidities (5), poor resilience (6), marital stress (7), and sub-scales of aggression (8). Through intensifying stress and anxiety, reducing sleep quality, and increasing the adoption of unhealthy behaviours, marital stress facilitates the incidence of depression (7). Moreover, aggression, particularly through anger and hostility pathways, intensifies the incidence of depression (8). So far, there has been little focus on the health literature on the mediator role of aggression in the relationship between underlying factors and depression. Therefore, the current study aims to evaluate and investigate the mediator role of aggression in the relationship between marital stress and depression.

## Materials and Methods

The population of current cross-sectional study includes all the patients with coronary artery disease (CAD) under coronary artery bypass graft (CABG) referred to the cardiac rehabilitation (CR) of the Imam Ali Hospital in Kermanshah City in Iran from Jan to Jun 2017. During the time frame of the study, 263 patients with CAD were participating in the CR, and after applying the inclusion criteria, 212 participants remained. The inclusion criteria for the study included fluency in the Persian language, the age range between 20 years to 80 years, and willingness to participate in the study. Moreover, incomplete answer sheets were eliminated from the study. In order to select the samples and gather the required data, at first, the qualified patients were identified by the research team. Then, the patients were entered into the study after completing written consent and get the necessary guarantees for the confidentiality of their identities. A day before the first exercise session of the CR programme, the demographic information (age, sex, education level, occupation, and marital status), medical records and risk factors (psychiatric history, personal and family history of CVDs, smoking, drug and alcohol abuse, hypertension, diabetes, hyperlipidemia, and body mass index), and the psychological data (depression, aggression, and marital stress) of the patients was gathered and recorded by the clinical psychologist and cardiologist of the CR research team. Beck’s Depression Inventory (BDI) questionnaire (9), Buss and Perry’s Aggression Questionnaire (BPAQ) (10), and Hudson’s Marital Satisfaction Index (HMSI) (11) were distributed among individual patients by the psychologist. Moreover, after the initial interview and examination, the cardiologist recorded the information related to risk factors as well as the medical history of patients in the research forms. The body mass indices (BMI) of patients were measured and recorded by the nutritionist.

### Research Instruments

BDI is a 21-item test of 3 scores for each item. The total score for this scale varies from 0 to 63. Beck et al. discovered the retest reliability in a one-week interval as 0.93 (9). Reliability and validity of this tool have been confirmed in the Iranian population (12).

BPAQ is a 29-item questionnaire where participants rank certain statements along with a 5-point continuum from ‘extremely opposed’ to ‘extremely agree’. The questionnaire has four sub-scales includes anger, hostility, verbal aggression and physical aggression. The scores are normalised on a scale of 0 to 1, with 1 being the highest level of aggression (10). This is a reliable and valid instrument for measuring Iranian samples (13).

HMSI is a 25-item scale that evaluates the severity of marital satisfaction/stress. Thirteen items of the scale are scored directly and 12 items are scored on the reverse. Answers are graded based on the Likert 5-point (rarely to most of the time). The positively worded items that were scored on the reverse are items of 1, 3, 5, 8, 9, 11, 13, 16, 17, 19, 20, 21 and 23. There is a higher score means a considerable marital conflict and stress. Cronbach’s alpha of the HMSI items is 0.96 which it is indicative of suitable reliability (11). Recently, this tool has been used well in Iranian cardiac patients (14).

### Statistical Analysis

Information on sex, education level, occupation, history of psychiatry diseases, personal and family history of heart diseases, smoking, drug addiction, alcohol consumption, hypertension, diabetes, hyperlipidemia, and body mass index (BMI) has been reported as a percentage and age, depression, aggression, and marital stress have been reported as the mean and standard deviation (SD). In the main analysis, the relationship between variables firstly was investigated using Pearson correlation coefficient. After reviewing and confirming the required statistical assumptions, including normality and the significance of the correlation between all variables (15), the structural equation method (SEM) was used to examine the main hypothesis. The analyses were performed using SPSS20 for Windows (IBM SPSS, Armonk, NY, USA) software and AMOS software. All tests were two-tailed and a *P*-value < 0.05 was considered as significant level.

## Results

The mean age of the samples was 58.5 ± 8.9 and 145 (68.4%) were male. Besides, 60.8% of participants had a BMI > 25. Other demographic factors and clinical data are shown in Table 1. Also, Table 2 shows the mean, standard deviation (SD) and a correlation coefficients matrix. As can be seen from this table, there is a significant relationship between all components together (*P* < 0.01).

Table 3 shows the results of path analysis to examine the role of mediator of aggression in the relationship between marital stress and depression. According to the results of this table, the standard effect coefficient of the marital stress pathway to aggression is 0.31 (*P* < 0.001). The standard effect coefficient of the aggression pathway to depression is 0.58 (*P* < 0.001). However, the direct effect of marital stress on depression is not significant (*P* = 0.586). Meanwhile, the results of the analysis indicate that variables of marital stress and aggression can simultaneously explain 35% of the variance of depression.

**Figure 1:**
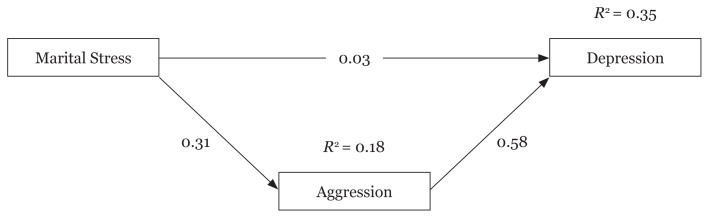
The research model of current study

The results of the path analysis indicate that aggression is a significant mediator in the relationship between marital stress and depression and the coefficient of indirect standard effect is 0.18. That is, marital stress is related to increased depression only when the intensity of aggression has increased (Figure 1).

## Discussion

The results of the current study show that marital stress cannot directly predict depression among patients with CAD. However, through aggression, marital stress can significantly predict increased depression. These findings are marginally confirmed by the previous studies (7, 8, 16–21). The obtained results will be discussed next.

It is believed that marital stress and conflict affects physical health through intense sympathetic excitation. Based on the Polyvagal theory, the vagal regulation, which is a measure of parasympathetic regulation, can intensify the consequences related to health caused by marital conflict context. Unhealthy chronic marital relations are often accompanied by endocrine changes, which can interfere with cardiovascular functions and the immune system (16). Concerning psychological health, chronic marital stress can lead to increased aggressiveness due to endocrine changes such as an elevation in the cortisol levels (17). Anger and hostility are factors related to marital conflict, which are often accompanied by hypertension and exaggerated physiological reactions such as variances in heart rate. Heart rate variances caused by marital stress play a significant role in verbal aggression towards the spouse. On the other hand, the hostility caused by unhealthy marital conflicts is related to a reduced prolactin and an increase in epinephrine, norepinephrine, and adrenocorticotrophin (16). Some of these neurotransmitters, such as norepinephrine, play an important role in cognitive and motivational regulation and social relations. Therefore, the interference of these neurotransmitters can lead to mood complications and depression (18).

Whitson and El-Sheikh (16) argue that marital stress is related to pessimism, including criticism, blame and chronic isolation. This factor can lead to rapid and consistent increases in epinephrine, norepinephrine and heart rate. Negative emotions and feelings such as aggression and each one of its components are the stimuli of the behavioural inhibition system (BIS). This system includes the septohippocampal system and its afferent monoaminergic neurons have spread from the brainstem and neocortical neoprene regions in the frontal lobe. BIS provides the motivational basis for behaviour inhibition, which may lead to undesired consequences. In other words, BIS can trigger physical processes and high-level cortex reactions and play an important role in emotional and cognitive reactions to environmental challenges (19). Recent studies (20, 21) show that there is a positive relationship between BIS and emotion regulation complications and the experience of increased depression. This pathway can justify the relationship between negative emotions such as aggression and depression.

The only question remained to be answered is why marital stress cannot directly predict depression. In order to answer this question, it can be said that all those suffering from marital conflicts are not necessarily reacting to their circumstances through the BIS. If in these individuals, the behavioural activation system (BAS) is the activating factor for thoughts and behaviours, the relationship between marital stress and depression is weakened. In this regard, Mellick et al. argue that there is no significant relationship between BAS and depression (21). Moreover, depression can be induced by several cognitive deviations such as blaming oneself for the conflicts with one’s spouse. However, in some cases, the spouses blame the other party for the problems and challenges. Because of this short-sightedness, the individual will feel anger and hostility towards his or her spouse, which may lead to aggressive behaviours. Finally, the individual’s resiliency level can be added to the raised considerations. Resilient people often show less depression (6).

In general, depression is one of the most common psychiatric predisposing factors and consequences of CVDs among CAD patients in the CR (1). Therefore, the search for its causes has always been of interest to the experts. Apparent and obvious predisposing factors related to depression such as personality traits, resilience, social support, and aggression in the cardiovascular patients have already been identified (4–6, 8) and have been involved in training related to control and reduce the risk factors of the disease. However, some underlying hidden causes such as marital conflicts indirectly exacerbate depression- as one of the most serious predisposing factors and outcomes of the CVDs. Depression, in turn, leads to a lack of adherence to CR (22). The consideration of this component in the development of modern CR protocols may enhance the benefits of these programs.

### Limitations

Similar to other studies in the field of medicine and behavioural sciences, this study also faced several limitations. In current study, only patients with CAD referred to CR were studied. Given that only certain groups referral to CR departments, the participation of other groups of cardiovascular patients in future studies can reduce the bias in the results. Due to a lack of fitness for the sub-scales of aggression (hostility, anger, verbal aggression, physical aggression), in the current model, only the total score of aggression was analysed. It is recommended that future studies select larger samples to be able to evaluate the mediator role of all the sub-components separately. Despite relatively similar the gender distribution and age range of the patients in the present study, some variables, such as psychiatry history, may potentially affect the results. However, controlling these variables and a greater number of covariates also requires a large sample. Although path analysis is a method for examining direct and indirect effects, it is intended not to discover causes. In fact, the aim of path analysis is an explanation, not a prediction (23). Although the present study model is not a complex framework, the assumptions must be carefully considered in future studies with more variables.

## Conclusion

Not directly, but in an indirect path through aggression, marital status can significantly predict increased depression among patients with CAD. Despite the basic constraints of path analysis and its inability to predict and discover the causes, in societies such as Iran, where marriage is emphasised and divorce is considered a social taboo, identifying poor consequences of the marital structure and its indirect pathways may help health policymakers for specific groups with CAD.

## Figures and Tables

**Table 1:** The demographic characteristics and medical history of the samples (*n* = 212)

Variables	*n* (%)
Education level	
Under diploma	124 (58.5)
Diploma	42 (19.8)
Academic education	46 (21.7)
Occupation	
Employed	25 (11.8)
Self-employed	58 (27.3)
Housekeeper	60 (28.3)
Retired	68 (32.1)
Unemployed	1 (0.5)
Medical history and risk factors	
Psychiatric history	58 (27.4)
CVDs in family	122 (57.5)
Experienced personal CVDs	36 (17)
Smoking	88 (41.5)
Drug addiction	22 (10.4)
Alcohol drinking	10 (4.7)
Hypertension	87 (41)
Diabetes	51 (24.1)
Hyperlipidemia	65 (30.7)

Abbreviation: CVDs = cardiovascular diseases

**Table 2:** Descriptive information of the samples and Pearson correlation coefficient matrix

Factors	Mean ± SD	Marital stress	Aggression	Depression
Marital stress	32.0 ± 14.06	1	**0.31**	**0.21**
Aggression	64.16 ± 18.88	**0.31**	1	**0.59**
Depression	16.41 ± 3.74	**0.21**	**0.59**	1

All values are as mean±standard deviation; Boldface indicates statistically significant ( *P* < 0.01)

**Table 3:** The results of path analysis

Paths	Direct effect			Indirect effect		
	
	B	*β*	*P*-value	B	*β*	*P*-value
Marital stress→Depression	0.01	0.03	0.586	0.06	0.18	< 0.001
Marital stress→Aggression	0.55	0.31	< 0.001	-	-	-
Aggression→Depression	0.12	0.58	< 0.001	-	-	-

B = regression weights, *β* = standardised regression weights
